# A Typology of Health Services Regulated in U.S. Assisted Living Communities

**DOI:** 10.1093/geroni/igab046.2028

**Published:** 2021-12-17

**Authors:** Paula Carder, Lindsey Smith, Wenhan Zhang, Sheryl Zimmerman, Philip Sloane, Kali Thomas

**Affiliations:** 1 OHSU-PSU School of Public Health, Portland, Oregon, United States; 2 Brown University, Providence, Rhode Island, United States; 3 Cecil G. Sheps Center for Health Services Research, Chapel Hill, North Carolina, United States; 4 UNC Medical School, Sheps Center, Chapel Hill, North Carolina, United States; 5 Brown University, Brown University/Providence, Rhode Island, United States

## Abstract

State agencies regulate assisted living (AL) with varying approaches across and within states. The implications of this variation for resident case mix, health service use, and policy, are not well described. We collected health services-relevant AL regulatory requirements for all 50 states and DC and used a mixed-methods approach (thematic analysis; k-means cluster analysis) to identify six types: Housing, Affordable, Hybrid, Hospitality, Healthcare, and Hybrid-Healthcare. We stratified Medicare claims data by regulatory type, identifying variation in resident case mix and health service use. Housing and Affordable clusters have larger proportions of dual-eligible beneficiaries, Black residents, and residents of Affordable had more long-term nursing home use compared to other clusters. Dual-eligible beneficiaries account for 26.6% of Housing cluster residents compared to 8.1% of Hybrid Healthcare cluster residents. We provide other examples and explain the implications in terms of sampling AL for single and multi-state studies, racial disparities, and health-related policies.

